# Benchmarking imputation strategies for missing time-series data in critical care using real-world-inspired scenarios

**DOI:** 10.1038/s41598-026-39035-z

**Published:** 2026-02-10

**Authors:** Michael Poette, Sandrine Mouysset, Daniel Ruiz, Vincent Pey, Jean-Marc Alliot, Vincent Minville

**Affiliations:** 1 Anaesthesia Critical Care and Perioperative Medicine Department, Toulouse University Hospital, Toulouse, France; 2 RESTORE, Inserm UMR1301, Toulouse University, Toulouse, France; 3 IRIT, CNRS UMR5505, Toulouse University, Toulouse, France

**Keywords:** Computer science, Data processing, Machine learning

## Abstract

Handling missing data remains a central challenge in Intensive Care Units (ICU) time-series analysis, where gaps frequently arise from non-random mechanisms such as sensor disconnections and workflow-driven interruptions. In this study, we benchmarked multiple imputation strategies on monitoring data from MIMIC-IV and designed masking scenarios that reflect ICU missingness patterns observed in the database, thereby approximating real-world conditions and clarifying how conclusions depend on both the chosen imputation method and the missingness scenario. We compared commonly used simple statistical approaches (mean, LOCF, interpolation), classical machine learning techniques (MICE, MissForest), and several deep learning architectures (Transformers, RNNs, GANs, VAEs). Transformer and GAN models achieved the best overall performance, whereas linear interpolation remained a strong baseline. Crucially, results were scenario-dependent: MCAR produced optimistic error estimates and compressed differences between methods, whereas structured gaps revealed clearer performance separations. Our findings suggest that, while deep learning methods improve overall imputation accuracy, linear interpolation is often nearly as effective and offers a lighter, more interpretable approach. This work introduces a practical framework for evaluating time-series imputation strategies under realistic constraints, with a focus on clinical relevance. Further analysis of downstream impact under clinically realistic scenarios and using tailored imputation strategies by variable type remains needed.

## Introduction

Patient monitoring in Intensive Care Units (ICUs) generates large volumes of time-series data, often characterized by high frequency and complexity. The rise of digital twin applications in healthcare has further reinforced the value of capturing temporal dynamics. Instead of relying solely on summary statistics over 24-hour periods (e.g., minimum, mean, or maximum), several studies have shown that analyzing trends over time can significantly enhance the prediction of critical outcomes ^1,2^.

Yet, a major obstacle remains: missing values are common in real-world ICU datasets. These gaps frequently result from non-random causes, including planned clinical actions (such as temporary transfer for procedures) or technical problems like sensor disconnections. If not handled properly, missing data can distort downstream analyzes and impair the performance of predictive models ^3–6^.

Simple approaches such as mean imputation or LOCF remain common but do not capture the temporal structure of multivariate ICU time series ^7–9^. More expressive sequence models (e.g., SAITS, BRITS) can model cross-temporal and cross-variable dependencies ^10,11^. Yet, outside benchmarking contexts, their robustness under real-life ICU missingness, often non-random, has not been firmly established ^12,13^.

Furthermore, benchmarking analyzes of imputation often involve heterogeneous data types, including continuous physiological signals, irregular laboratory measurements, and discrete clinical events ^9,10,14^. These data exhibit distinct temporal resolutions and acquisition processes in the ICU, and their missingness should be addressed with methods tailored to the underlying physiology and the Electronic Health Record (EHR) workflow. Otherwise, benchmarking results may be misleading.

In this light, continuous bedside monitoring variables such as Heart Rate (HR), Oxygen Saturation (SpO$_2$), Respiratory Rate (RR), and Mean Blood Pressure (MBP) might be prime candidates for more complex imputation strategies, given their continuous nature, and potential cross-variable dependencies.

In this study, we benchmark a broad panel of imputation methods, ranging from basic statistical techniques to advanced deep learning models, on realistic ICU time series. Our objectives are twofold: (i) to determine whether methods remain reliable and suitable for clinical deployment under realistic, non-random ICU missingness scenarios; and (ii) to evaluate their performance using clinically interpretable metrics.

## Dataset and preliminary analysis

## Study design and ethics

This retrospective study uses de-identified ICU data from the publicly available MIMIC-IV database ^15^. All records are de-identified in accordance with the Health Insurance Portability and Accountability Act (HIPAA), and access was granted under the PhysioNet Credentialed Health Data Use Agreement. Institutional Review Board (IRB) approval and informed consent were therefore not required.

### Cohort selection and variables

We included adult MIMIC-IV patients with ICU length of stay $\ge$48 h and extracted a 48 h window starting at ICU admission. An ICU stay was defined as a single contiguous intensive-care episode: intra-hospital transfers between ICU locations were considered part of the same stay when there was no transition to non-ICU care and no temporal gap between locations; any ICU readmission after discharge (or any intervening non-ICU interval) constituted a new stay. We excluded patients $<18$ years and ICU stays $<48$ h.

We focused on four routinely and continuously monitored variables: Heart Rate (HR), Oxygen Saturation (SpO$_2$), Respiratory Rate (RR), and Mean Blood Pressure (MBP), leveraging their continuous sampling to compare simple interpolation baselines with more expressive sequence models. Continuous monitoring is central to our benchmarking objective: these signals display mechanism-relevant missingness (short intermittent gaps, sensor-specific dropouts, prolonged interruptions), enabling a fair and informative evaluation. These variables are clinically pivotal in critical care analysis  ^16^ and widely available across ICU databases.

To remove implausible values, each variable was restricted to within $\pm 3$ standard deviations of its mean, with means and standard deviations computed on the training split only. Additional physiological plausibility filters were applied (e.g., negative blood pressure or SpO$_2$ values above 100% were discarded). Time intervals were aggregated on an hourly basis, an approach aligned with common ICU EHR storage conventions (including MIMIC) and widely adopted in the literature; if multiple measurements occurred within an hour, we used the median. Hours without any recorded measurement were considered missing. We excluded stays with more than 16 missing hourly values ($\approx$33% of the window), a threshold chosen a priori to balance cohort size and imputability. Data were standardized using parameters fitted on the training split only.

### Demographic and time-series overview

Table 1 summarizes the demographic characteristics of the final cohort, which included 26,167 ICU stays. The mean age was 63.5 years (SD = 16.6), with 55.2% of patients identified as male. The in-ICU mortality rate was 16.2%, and the average length of ICU stay (LOS) was 6.3 days (SD = 6.3), illustrating the clinical heterogeneity of the cohort.

Descriptive statistics for the four selected variables are presented in Table 2. HR, RR, and MBP were approximately normally distributed, while SpO$_2$ exhibited a skew toward higher saturation values.

**Fig. 1:**
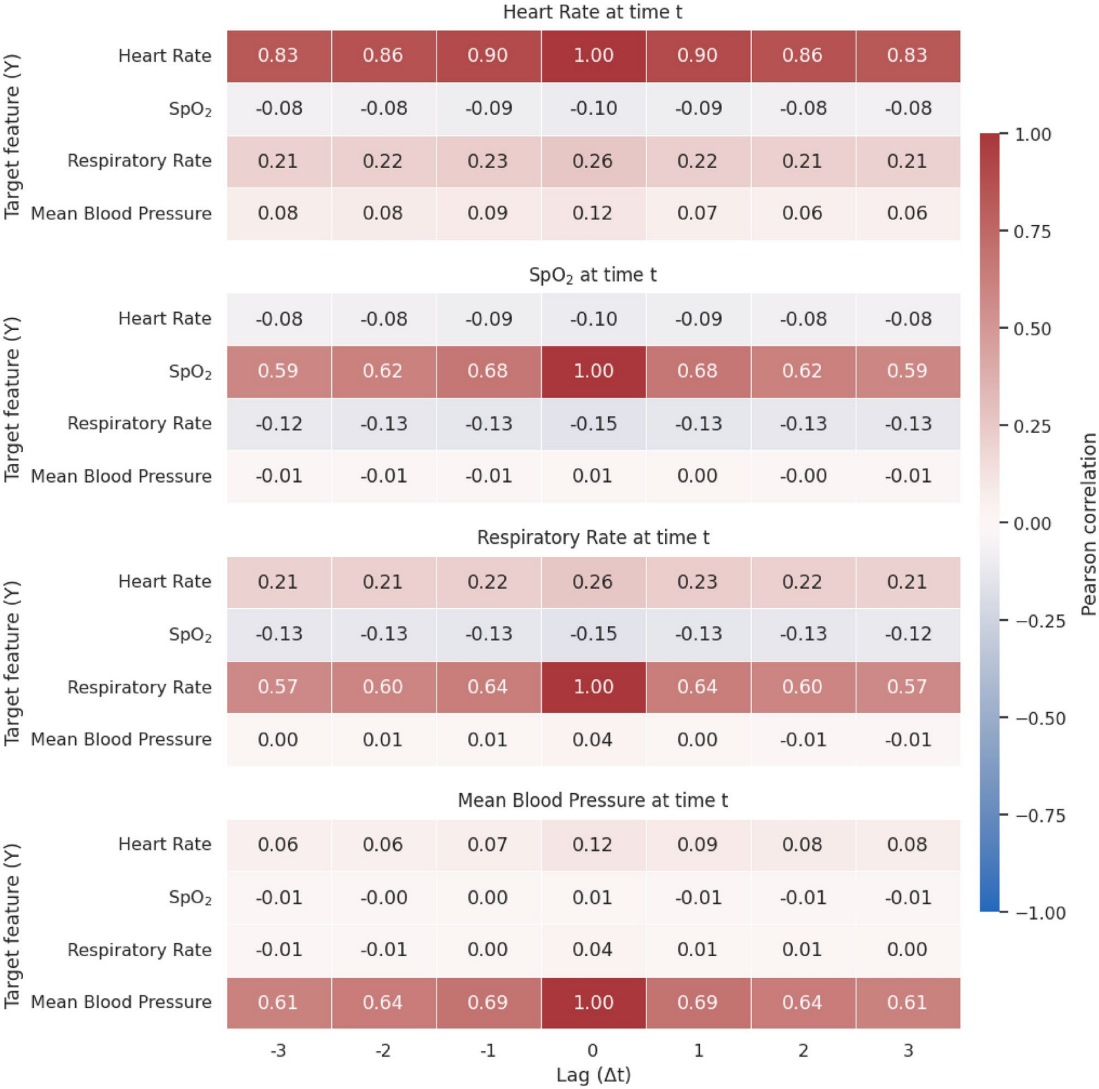
Lagged Pearson correlations across ICU vital signs for each timestamps with a time delta (lag) comprise between $-3$ to +3 h.

To characterize dependence structure beyond same-timestamp associations, Fig. 1 reports lagged Pearson correlations for short lags ($\Delta t\in [-3,3]$ h). Off-diagonal (cross-feature) correlations were uniformly low, including at zero lag, indicating limited instantaneous redundancy between vital signs. By contrast, within-feature autocorrelations were high across short lags, with heart rate exhibiting the strongest persistence (reaching 0.90 at $\Delta t=0$ and remaining $\ge$ 0.8 up to $\pm 3$ h).

**Table 1:** Demographic characteristics of the selected patients from MIMIC-IV dataset. *BMI* body mass index, *LOS* length of stay, *SAPS* simplified acute physiology score.

Number of ICU stays		26167
Age (*mean*, *SD*)		63.5 (16.6)
Gender ($n, \%$)	Male	14,455 (55.2)
BMI (*mean*, *SD*)		28.9 (8.3)
LOS (*mean*, *SD*)		6.3 (6.3)
Admission type ($n, \%$)	Medical	20,357 (77.8)
	Scheduled surgery	221 (0.8)
	Unscheduled surgery	5589 (21.4)
SAPS II (*mean*, *SD*)		38.5 (13.9)
Death ($n, \%$)		4245 (16.2)

**Table 2:** Temporal features statistic.

Statistic	Heart rate	SpO$_2$	Respiratory rate	Mean blood pressure
	(bpm)	(%)	(/min)	(mmHg)
count	1,221,710	1,204,610	1,211,796	1,193,046
mean	86.6	96.9	19.6	78.1
SD	18.4	2.6	5.4	14.5
Min	31	88	3	29
Q1	73	95	16	68
Median	85	97	19	76
Q3	99	99	23	87
Max	143	100	37	132

### Initial missingness profiling

Overall, missing values accounted for 3.8% of all entries in the dataset. At the feature level, missingness rates were 3.5% (n = 44,220) for respiratory rate, 2.7% (n = 34,306) for heart rate, 4.1% (n = 51,406) for SpO$_2$, and 5.0% (n = 62,970) for mean blood pressure.

**Fig. 2:**
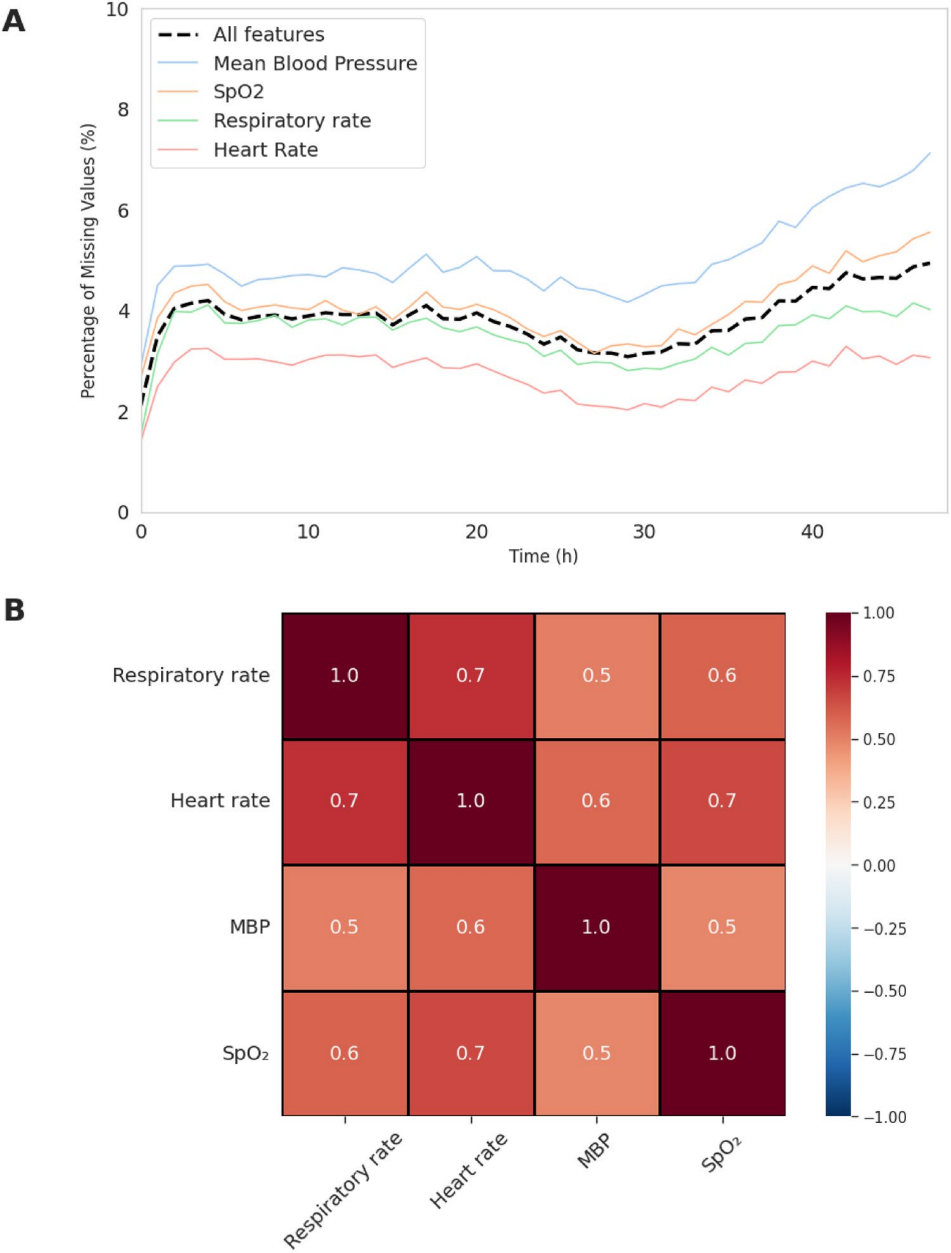
Representation of missingness patterns. **(A)** Percentage of missing values over hourly intervals. The dotted black line indicates the overall percentage of missing values across all features at each time point, while separate colored curves represent the percentage of missing values for individual variables.**(B)** Heatmap of nullity correlation, calculated using Pearson’s coefficient, ranging from $-1$ (inverse co-occurrence) to $+1$ (perfect co-occurrence).

The analysis of missing-data patterns (Fig. 2) showed a tendency for gaps to accumulate between 30 and 48 h after ICU admission. Moderate-to-strong pairwise nullity correlations across variables support the presence of shared missingness mechanisms. Most missing segments were short: 53% lasted a single hour and affected only one variable. In contrast, 18% and 7% of gaps spanned two and three consecutive hours, respectively. Around 16% of missing intervals involved all four variables simultaneously. Roughly 3% of cases exhibited longer gaps of up to four consecutive hours, generally confined to a single variable. Detailed distributions of missing features per timestamp and lengths of consecutive missing segments are provided in the Supplementary Material (Figs. S1 and S2).

These structured patterns are consistent with plausible real-world explanations, such as brief monitoring suspensions during procedures or transient sensor failures. Collectively, the findings suggest that missingness in this dataset is not random but instead reflects systematic clinical or technical factors. Accordingly, we constructed three masking scenarios that closely mirror the observed missingness patterns, ensuring that the benchmark reflects clinically realistic conditions.

## Real-world-inspired masking scenarios

Based on the missingness patterns identified in our preliminary analysis, we defined three masking strategies that collectively remove approximately 30% of the available data. These scenarios were designed to reflect both random and structured mechanisms commonly observed in ICU settings:

- Random missingness (MCAR): data points are independently removed across time and variables, emulating sporadic, isolated gaps. While this setup is widely used in the literature, it also matches real cases in our dataset where a single variable is occasionally missing in a given hour.
- Temporal interruptions: periods of 1 to 3 consecutive hours are masked simultaneously across all four variables. This mimics real-world situations like patient transport for imaging or procedures, during which all monitoring may be temporarily suspended.
- Sensor failures (single variable): one variable is removed over a continuous 4-hour window, representing isolated sensor outages such as SpO$_2$ probe displacement or arterial line issues.

These scenarios were applied to the standardized dataset for both training and testing phases. As detailed below, they introduce distinct imputation challenges, from isolated random gaps to longer, structured blocks, enabling a comprehensive assessment of robustness across mechanisms. We trained models under MCAR masks to avoid injecting mechanism-specific priors and to assess out-of-distribution robustness. Evaluation covered three mechanisms: MCAR, temporal interruptions (contiguous gaps), and sensor-specific masking.

## Selection and implementation of imputation methods

After identifying realistic missing data patterns, we evaluated a broad range of imputation techniques, from simple statistical baselines to advanced deep learning models. Our aim was to compare their performance across both random and structured missingness scenarios.

Statistical and traditional methods:

- Mean/median imputation: missing values are replaced by the mean or median of observed values for each patient-variable pair. While easy to implement, these methods ignore temporal dynamics and may introduce bias.
- LOCF (last observation carried forward): each missing value is filled using the most recent non-missing observation. This technique is common in clinical settings but may distort temporal trends if missing segments are long.
- Linear interpolation: estimates missing values by connecting the nearest available observations with a straight line. Although univariate, it performs well when data are relatively stable or inter-variable dependencies are weak.

Machine learning-based approaches:

- MICE (multiple imputation by chained equations): iteratively models each variable with missing data based on all others. Originally intended for tabular datasets, it requires reshaping time-series into a flat format.
- MissForest: uses random forests to iteratively impute missing values. It better captures non-linear relations than MICE but similarly discards temporal structure by working on reshaped data.

Deep learning-based methods:

- SAITS (self-attention-based imputation for time series): a transformer-based model that leverages self-attention to capture long-range temporal dependencies ^10^.
- BRITS (bidirectional recurrent imputation for time series): employs forward and backward recurrent neural networks to infer values from both past and future contexts ^11^.
- US-GAN (unsupervised sequential generative adversarial network): applies a GAN-based framework to generate plausible imputations that align with the distribution of observed data ^17^.
- GP-VAE (Gaussian process variational autoencoder): combines Gaussian processes with a variational autoencoder to generate probabilistic imputations ^18^.

All deep learning models were implemented using the PyPOTS library (https://github.com/WenjieDu/PyPOTS). Hyperparameters were tuned via grid search on a 20% validation split, and the selected configurations were trained for 200 epochs. Data were standardized before training, and imputed values were subsequently back-transformed to the original units for clinical interpretability. Full details of the training protocol and the hyperparameter grids are provided in the Supplementary Material (Section S2; Tables S1–S2).

The dataset was split into training, validation, and test sets. For all models, training was conducted using the same data where 30% of values were masked under a random (MCAR) pattern. This reflects a realistic scenario where the missingness mechanism at inference time is unknown, and enables fair assessment of each model’s generalization ability.

Final evaluation was conducted on the test set under the predefined masking scenarios (MCAR, temporal interruption, sensor-specific). Imputation accuracy was quantified with Mean Absolute Error (MAE) and Root Mean Squared Error (RMSE) defined by Eq. (1) and computed only on masked entries:

1 $$\begin{aligned} MAE = \frac{1}{n}\sum _{i=1}^{n} |x_i - y_i|, \hspace{2cm} RMSE = \sqrt{ \frac{1}{n}\sum _{i=1}^{n} (x_i - y_i)^2 }. \end{aligned}$$

Metrics are reported on standardized scales and, when relevant, back-transformed to physical units. Results are averaged over 5 random seeds encompassing both mask construction and any stochastic components of the imputers; we report mean±SD across runs.

## Benchmarking results across masking scenarios

We report aggregated MAE/RMSE on standardized data, then analyzes focused on MBP in physical units (mmHg).

### Overall performance (standardized metrics)

We first report standardized MAE across all monitored variables and masking strategies (Table 3). Overall, neural network-based approaches, particularly SAITS and US-GAN, outperformed traditional statistical methods. Surprisingly, linear interpolation also showed strong results, with performance approaching that of the more complex models. This likely reflects the strong proximal temporal continuity in ICU monitoring data.

By contrast, more sophisticated statistical methods such as MissForest and MICE consistently underperformed, even relative to simpler techniques. One explanation is that converting time-series data into tabular form, as required by these methods, disrupts temporal structure and hampers their ability to model time-dependent patterns accurately.

Within the deep learning category, architecture played a key role: Transformer-based models (like SAITS) and generative adversarial network models (like US-GAN) outperformed RNN-based models (e.g., BRITS) and VAE-based models (e.g., GP-VAE). The self-attention mechanism in Transformers likely enabled better capture of long-range temporal and cross-variable dependencies.

We also observed pronounced, scenario-dependent differences. Randomly distributed missingness (MCAR) consistently yielded the lowest errors, whereas *structured* gaps, particularly 1–3-h consecutive blocks, were markedly harder. Error increased monotonically with gap length: short, contiguous gaps were largely recoverable, but extending the same block by even one hour produced a disproportionate rise in both MAE and RMSE. Under single-variable masking, performance depended strongly on the intrinsic predictability of the signal; for example, heart rate remained comparatively predictable even when disrupted for 4 hours, while less autocorrelated variables degraded more steeply. Across scenarios, SAITS, US-GAN, and linear interpolation were the most resilient for short-term gaps; however, as blocks lengthened, SAITS and US-GAN degraded more gracefully than interpolation, suggesting benefits from modeling long-range temporal and cross-variable dependencies. Yet, the consistency of linear interpolation across variables suggests that local temporal continuity often matters more than multivariate correlations.

The comparison of MAE and RMSE (Table 4) revealed systematic discrepancies: RMSE values were consistently higher than MAE, indicating the presence of outliers or high-variance errors. These differences were most pronounced for simpler methods (e.g., mean imputation, LOCF) and for MissForest or MICE, suggesting a greater tendency toward large deviations. In contrast, SAITS and linear interpolation showed smaller MAE/RMSE gaps, implying better stability and fewer extreme errors.

Taken together, these findings suggest that although neural models offer improved average performance, linear interpolation remains a surprisingly strong and interpretable baseline, especially for short gaps. Methods that lack explicit temporal modeling (e.g., MissForest, MICE) appear less suitable for ICU time-series imputation.

### Clinical relevance

To better evaluate potential bedside impact, we focused on imputing missing MBP values, a vital sign with direct clinical relevance to hemodynamic management. For each method and scenario, we extracted the masked MBP values, reconstructed them, and then back-transformed the errors to physical units (mmHg). MAE and RMSE results in mmHg are reported in Tables 5 and 6.

Trends across methods mirrored the standardized results. For short missing blocks (1–2 h), most methods kept errors within single-digit mmHg. In such cases, differences between SAITS, US-GAN, and simpler approaches like linear interpolation were modest, often around 2–3 mmHg, and unlikely to be clinically meaningful. Even under more challenging conditions (e.g., prolonged sensor failure), most methods maintained RMSE values within 10–15 mmHg, a range typically considered acceptable.

While advanced neural models often achieved the lowest errors, the actual gains in mmHg were sometimes small, emphasizing that superior standardized metrics do not always equate to meaningful clinical differences.

**Fig. 3:**
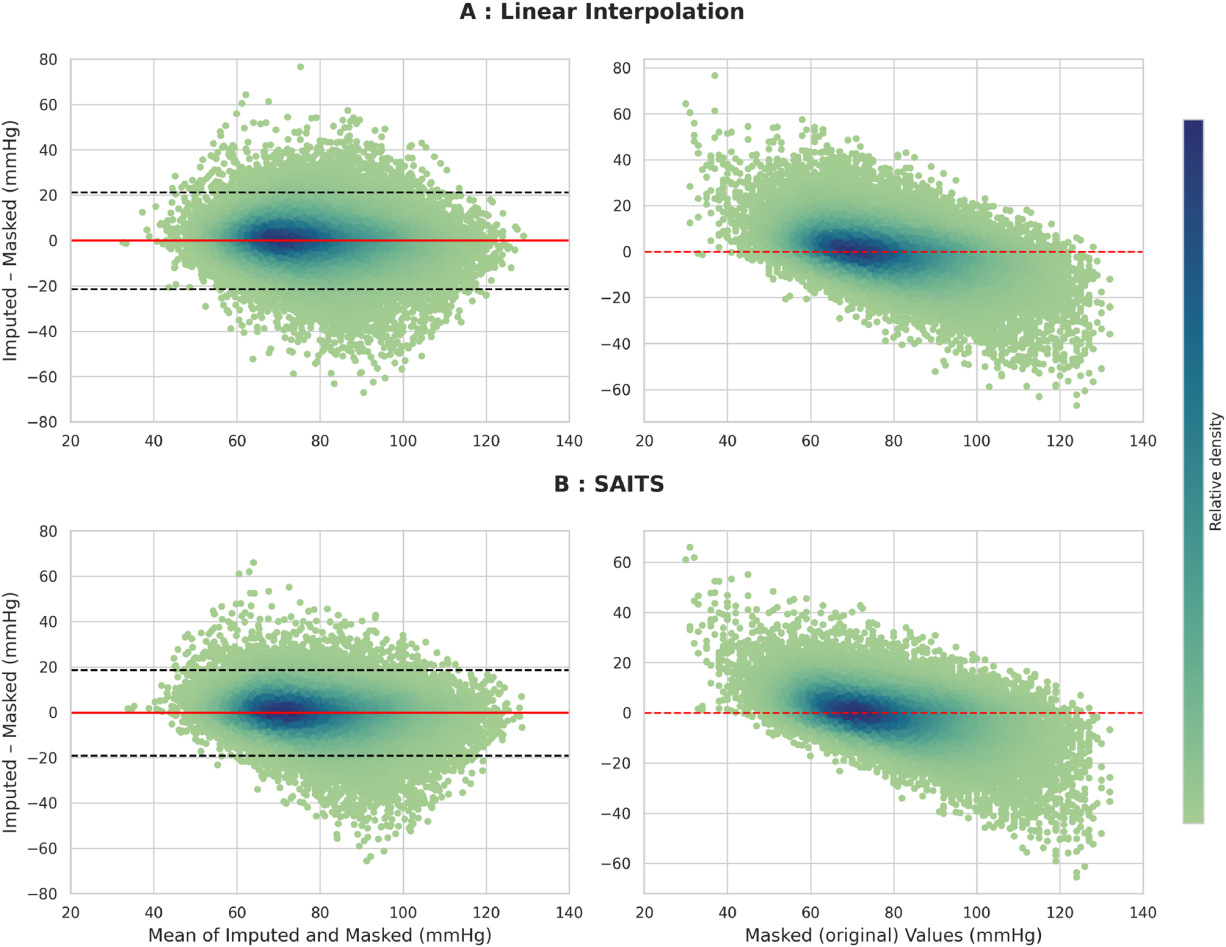
Distribution of imputation errors for mean arterial pressure (MAP) using linear interpolation (**A**) and SAITS (**B**). Each point represents a masked measurement ($n = 44{,}100$), with relative density encoded by colour intensity. Left panels show Bland-Altman plots, where the *x*-axis represents the average of imputed and masked values, and the *y*-axis their difference. The red line indicates the mean bias, and the dashed black lines show the 95% limits of agreement ($\pm 1.96 \times \text {SD}$). Right panels display standard residual plots, with imputation error ($\text {imputed} - \text {masked}$) plotted against the original masked values.

To assess performance across the physiological spectrum, we plotted Bland–Altman and residual plots for MBP (Fig. 3). Bland-Altman analysis, commonly used in clinical research, evaluates the agreement between two measurement methods. Residual plots, by contrast, illustrate how errors vary with respect to the true values. Together, these visualizations provide complementary insights, especially when ground-truth data may include measurement noise or edge cases.

Both SAITS and linear interpolation exhibited low bias and narrow limits of agreement in the mid-physiological range (60–90 mmHg), indicating reliable performance for most ICU patients. However, performance deteriorated at physiologic extremes. For MBP > 100 mmHg ($n = 3{,}499$, 3.7%), RMSE rose to 16.7 mmHg for SAITS and 17.3 mmHg for linear interpolation. Similarly, for MBP < 65 mmHg ($n = 1{,}309$, 1.4%), RMSE reached 15.2 mmHg and 16.5 mmHg, respectively. These rare but clinically critical situations contributed disproportionately to overall error and reflect the vulnerability of current methods under edge conditions.

In summary, while neural models like SAITS and US-GAN show measurable improvements, the clinical relevance of these gains depends heavily on the patient’s physiological state. This highlights the importance of imputation strategies that remain reliable precisely when clinical decisions are most sensitive to error.

**Table 3:** MAE across five random seeds (mean ± SD) for each imputation method under eight masking strategies. Lower is better; best per column in bold.

		All features missing			Single-variable 4-hours			
	Random missing	1-hour block	2-hours block	3-hours block	RR only	HR only	MBP only	SpO$_2$ only
Mean	0.952 $\pm \,0.014$	0.939 $\pm \,0.014$	0.963 $\pm \,0.021$	0.938 $\pm \,0.017$	0.854 $\pm \,0.012$	0.845 $\pm \,0.035$	1.319 $\pm \,0.052$	0.840 $\pm \,0.019$
Median	0.943 $\pm \,0.018$	0.936 $\pm \,0.024$	0.946 $\pm \,0.034$	0.931 $\pm \,0.014$	0.902 $\pm \,0.002$	0.872 $\pm \,0.118$	1.277 $\pm \,0.104$	0.826 $\pm \,0.009$
Linear interp.	0.445 $\pm \,0.004$	0.438 $\pm \,0.001$	0.475 $\pm \,0.001$	0.460 $\pm \,0.002$	0.570 $\pm \,0.001$	0.306 $\pm \,0.002$	0.541 $\pm \,0.003$	0.538 $\pm \,0.003$
LOCF	0.520 $\pm \,0.005$	0.513 $\pm \,0.001$	0.561 $\pm \,0.002$	0.541 $\pm \,0.003$	0.654 $\pm \,0.004$	0.392 $\pm \,0.003$	0.642 $\pm \,0.002$	0.623 $\pm \,0.007$
MICE	0.780 $\pm \,0.001$	0.796 $\pm \,0.002$	0.795 $\pm \,0.001$	0.797 $\pm \,0.001$	0.752 $\pm \,0.001$	0.757 $\pm \,0.001$	0.776 $\pm \,0.004$	0.811 $\pm \,0.004$
MissForest	1.141 $\pm \,0.002$	1.092 $\pm \,0.001$	1.090 $\pm \,0.003$	1.093 $\pm \,0.002$	1.182 $\pm \,0.004$	1.221 $\pm \,0.009$	1.057 $\pm \,0.005$	1.156 $\pm \,0.006$
SAITS	**0.430** $\pm \,0.004$	0.435 $\pm \,0.001$	**0.462** $\pm \,0.001$	**0.450** $\pm \,0.001$	**0.499** $\pm \,0.002$	**0.297** $\pm \,0.002$	**0.478** $\pm \,0.002$	0.552 $\pm \,0.001$
BRITS	0.609 $\pm \,0.008$	0.666 $\pm \,0.002$	0.678 $\pm \,0.001$	0.673 $\pm \,0.001$	0.604 $\pm \,0.002$	0.388 $\pm \,0.002$	0.739 $\pm \,0.004$	0.683 $\pm \,0.004$
US-GAN	0.433 $\pm \,0.006$	**0.433** $\pm \,0.001$	0.483 $\pm \,0.001$	0.460 $\pm \,0.001$	0.537 $\pm \,0.001$	0.413 $\pm \,0.002$	0.502 $\pm \,0.003$	**0.523** $\pm \,0.002$
GP-VAE	0.743 $\pm \,0.003$	0.741 $\pm \,0.002$	0.774 $\pm \,0.001$	0.767 $\pm \,0.001$	0.760 $\pm \,0.001$	0.727 $\pm \,0.001$	0.777 $\pm \,0.004$	0.817 $\pm \,0.004$

**Table 4:** RMSE across five random seeds (mean ± SD) for each imputation method under eight masking strategies. Lower is better; best per column in bold.

	Random missing	All features missing			Single-variable only			
		1-hour block	2-hours block	3-hours block	RR only	HR only	MBP only	SpO$_2$ only
Mean	1.162 $\pm \,0.015$	1.145 $\pm \,0.021$	1.143 $\pm \,0.019$	1.176 $\pm \,0.028$	1.046 $\pm \,0.011$	1.034 $\pm \,0.034$	1.523 $\pm \,0.054$	1.018 $\pm \,0.009$
Median	1.161 $\pm \,0.024$	1.147 $\pm \,0.033$	1.142 $\pm \,0.017$	1.163 $\pm \,0.047$	1.096 $\pm \,0.001$	1.075 $\pm \,0.126$	1.479 $\pm \,0.108$	1.038 $\pm \,0.040$
Imputation avg.	0.657 $\pm \,0.004$	0.647 $\pm \,0.001$	0.673 $\pm \,0.003$	0.690 $\pm \,0.002$	0.795 $\pm \,0.003$	0.445 $\pm \,0.005$	0.744 $\pm \,0.003$	0.778 $\pm \,0.006$
MICE	0.972 $\pm \,0.001$	0.990 $\pm \,0.002$	0.991 $\pm \,0.001$	0.987 $\pm \,0.001$	0.955 $\pm \,0.002$	0.941 $\pm \,0.002$	0.975 $\pm \,0.005$	0.990 $\pm \,0.004$
MissForest	1.405 $\pm \,0.002$	1.352 $\pm \,0.001$	1.354 $\pm \,0.002$	1.350 $\pm \,0.004$	1.427 $\pm \,0.005$	1.518 $\pm \,0.010$	1.332 $\pm \,0.005$	1.394 $\pm \,0.007$
SAITS	**0.605** $\pm \,0.004$	0.612 $\pm \,0.002$	**0.630** $\pm \,0.002$	**0.641** $\pm \,0.002$	**0.686** $\pm \,0.003$	**0.425** $\pm \,0.005$	**0.654** $\pm \,0.002$	0.719 $\pm \,0.002$
BRITS	0.808 $\pm \,0.008$	0.871 $\pm \,0.003$	0.878 $\pm \,0.001$	0.881 $\pm \,0.001$	0.780 $\pm \,0.003$	0.507 $\pm \,0.004$	0.942 $\pm \,0.005$	0.861 $\pm \,0.005$
US-GAN	0.608 $\pm \,0.006$	**0.605** $\pm \,0.002$	0.632 $\pm \,0.002$	0.654 $\pm \,0.002$	0.723 $\pm \,0.001$	0.535 $\pm \,0.002$	0.667 $\pm \,0.003$	**0.712** $\pm \,0.003$
GP-VAE	0.932 $\pm \,0.004$	0.930 $\pm \,0.002$	0.956 $\pm \,0.001$	0.963 $\pm \,0.001$	0.964 $\pm \,0.002$	0.903 $\pm \,0.002$	0.975 $\pm \,0.005$	0.994 $\pm \,0.004$

**Table 5:** Mean absolute error (mmhg) for mean blood pressure under five masking strategies, reported as mean ± SD over five seeds. Lower is better; best per column in bold.

	Random	All features missing			
		1-hour block	2-hour block	3-hour block	MBP only
Mean	19.22 $\pm \,0.54$	18.55 $\pm \,0.91$	18.31 $\pm \,0.88$	19.71 $\pm \,1.23$	19.27 $\pm \,0.76$
Median	18.38 $\pm \,0.51$	18.07 $\pm \,1.22$	17.22 $\pm \,0.99$	18.42 $\pm \,1.64$	18.65 $\pm \,1.52$
Linear interp.	7.26 $\pm \,0.06$	7.17 $\pm \,0.02$	7.47 $\pm \,0.02$	7.69 $\pm \,0.05$	7.90 $\pm \,0.04$
MICE	11.37 $\pm \,0.05$	11.41 $\pm \,0.06$	11.41 $\pm \,0.02$	11.36 $\pm \,0.03$	11.33 $\pm \,0.06$
MissForest	14.71 $\pm \,0.15$	11.76 $\pm \,0.04$	11.77 $\pm \,0.02$	11.72 $\pm \,0.03$	15.43 $\pm \,0.08$
SAITS	**6.64** $\pm \,0.06$	**6.71** $\pm \,0.02$	**6.94** $\pm \,0.01$	**7.09** $\pm \,0.04$	**6.97** $\pm \,0.03$
BRITS	10.96 $\pm \,0.05$	11.22 $\pm \,0.06$	11.22 $\pm \,0.02$	11.19 $\pm \,0.04$	10.79 $\pm \,0.06$
US-GAN	6.80 $\pm \,0.09$	6.92 $\pm \,0.02$	7.36 $\pm \,0.01$	7.72 $\pm \,0.02$	7.32 $\pm \,0.04$
GP-VAE	11.38 $\pm \,0.05$	11.38 $\pm \,0.06$	11.40 $\pm \,0.02$	11.35 $\pm \,0.03$	11.34 $\pm \,0.06$

**Table 6:** Root mean squared error (mmHg) for mean blood pressure under five masking strategies, reported as mean ± SD over five seeds. Lower is better; best per column in bold.

	Random	All features missing			
		1-hour block	2-hours block	3-hours block	MBP only
Mean	22.19 $\pm \,0.56$	21.50 $\pm \,0.96$	21.24 $\pm \,0.93$	22.68 $\pm \,1.27$	22.24 $\pm \,0.79$
Median	21.32 $\pm \,0.53$	21.00 $\pm \,1.27$	20.10 $\pm \,1.04$	21.34 $\pm \,1.71$	21.60 $\pm \,1.58$
Linear interp.	10.14 $\pm \,0.07$	10.02 $\pm \,0.03$	10.39 $\pm \,0.03$	10.63 $\pm \,0.06$	10.87 $\pm \,0.05$
MICE	14.30 $\pm \,0.06$	14.31 $\pm \,0.06$	14.33 $\pm \,0.03$	14.27 $\pm \,0.05$	14.24 $\pm \,0.07$
MissForest	18.58 $\pm \,0.17$	14.74 $\pm \,0.04$	14.74 $\pm \,0.04$	14.70 $\pm \,0.06$	19.45 $\pm \,0.07$
SAITS	**9.24** $\pm \,0.06$	**9.35** $\pm \,0.04$	**9.60** $\pm \,0.02$	**9.77** $\pm \,0.07$	**9.56** $\pm \,0.03$
BRITS	13.99 $\pm \,0.06$	14.29 $\pm \,0.06$	14.31 $\pm \,0.03$	14.27 $\pm \,0.06$	13.76 $\pm \,0.07$
US-GAN	9.27 $\pm \,0.09$	9.36 $\pm \,0.04$	9.77 $\pm \,0.02$	10.10 $\pm \,0.04$	9.73 $\pm \,0.04$
GP-VAE	14.29 $\pm \,0.06$	14.26 $\pm \,0.06$	14.29 $\pm \,0.03$	14.24 $\pm \,0.05$	14.23 $\pm \,0.07$

## Discussion

In line with prior studies, our results validate the effectiveness of Transformer-based architectures (SAITS) and Generative Adversarial Network models (US-GAN) for time-series imputation, particularly when data are missing at random or in short blocks, with US-GAN performing comparably to SAITS. Their mechanisms capture long-range dependencies, leading to strong performance in scenarios featuring scattered gaps. However, they displayed higher error rates at physiological extremes, possibly because such rare out-of-range samples are underrepresented during training. Clinically, this limitation is nontrivial, as extreme vitals may signal severe pathophysiological states that demand accurate monitoring.

Meanwhile, linear interpolation emerged as a robust alternative, delivering near-comparable performance in many settings and demonstrating a stable error distribution, even at physiological outliers. We hypothesize that the relatively weak cross-variable correlations in these ICU signals may limit the added value of complex multivariate models in such contexts. This interpretation is supported by the lagged-correlation analysis (Fig. 1), which shows uniformly low cross-feature correlations, including at zero lag, contrasted with strong within-feature autocorrelation. Such a dependence structure favors proximal univariate continuity and helps explain why interpolation performs competitively for short gaps. Moreover, linear interpolation’s simplicity, low computational cost, and transparency make it especially attractive for deployment in resource-constrained settings or embedded applications.

Several other methods yielded less favorable results:

- Mean Imputation: consistently underperformed, reinforcing that ignoring temporal structure introduces bias.
- MICE and MissForest: though more sophisticated than naive methods, their reliance on tabular assumptions increased computational burden without notable performance benefits in our use case.
- BRITS, GP-VAE: these deep learning models captured temporal dependencies but varied in performance depending on their architecture. BRITS, based on bidirectional RNNs, came closest to SAITS, whereas GP-VAE performed less consistently. Hybrid models like BRATI ^19^, combining attention and recurrence, offer promising directions for future work.

Across five independent seeds, all imputation methods were *stable*, with low standard deviations that did not alter method rankings.

By designing masking scenarios based on observed ICU missingness patterns, we aimed to bridge the gap between synthetic benchmarks and real-world clinical conditions. Our dataset, drawn from over 26,000 ICU stays and covering a clinically relevant 48-h window, provides a robust foundation for evaluating imputation methods. Scenario effects were pronounced: MCAR consistently underestimated error relative to structured block-missingness (e.g., 1–3-h consecutive gaps), making MCAR an optimistic lower bound rather than a realistic expectation of clinical performance. This suggests that absolute MCAR benchmarking results on mixed feature types (continuous monitoring, laboratory results, therapies/medications, etc.) should be interpreted with caution and do not reflect the quality itself of the data imputed on missing values.

Several limitations merit consideration. We deliberately focused on continuously monitored physiological variables and did not model discrete events (e.g., medication administrations) or clinical interventions (e.g., mechanical ventilation), which can influence both sampling and missingness. We adopted an hourly time step because it is the cadence most commonly emphasized in ICU EHR time-series analyzes and the most readily available across databases; coarser resolutions (e.g., daily) or finer ones (minutes to milliseconds, waveform-level) have different statistical properties and would require dedicated preprocessing, modeling, and validation pipelines. This scoped design improves methodological clarity for imputation of continuous streams, but it also means that many clinically meaningful downstream tasks (e.g., risk modeling, event prediction) would require a broader multimodal feature set (e.g., laboratory results, therapies) and task-specific ground truths: from a multi-phase perspective, analysis validity further hinges on the congeniality between imputation and the downstream model ^20^. In addition, because native data already contain gaps, long-horizon targets such as time-under-threshold cannot be validated without additional assumptions. Accordingly, we prioritize robust imputation under realistic mechanisms as a foundation and leave multimodal, task-specific downstream evaluations for future work.

It is also important to consider the nature of the “ground truth” used in this evaluation. In the MIMIC-IV database, reference values, particularly for blood pressure, may originate from diverse measurement modalities: non-invasive cuffs, invasive arterial lines (radial or femoral), or sporadic readings during imaging procedures. Each technique carries its own level of precision and susceptibility to artefacts. In this light, our use of Bland-Altman plots is especially appropriate, as this method does not assume a perfect reference but rather assesses agreement between two measurement sources. For clinical datasets with inherent measurement variability, such visualizations may provide more meaningful insights than global accuracy metrics alone.

Although MAE and RMSE are intuitive and widely used, they can obscure systematic biases or errors in clinically sensitive ranges. Our Bland-Altman analysis showed increased variance and bias at physiological extremes, where small imputation errors may have disproportionate consequences. Recent works have highlighted the limitations of these classical metrics and proposed alternatives such as distributional scores or clinical-error thresholds ^21^. Some even suggest bypassing imputation entirely by modeling missingness as an informative feature, as in the approach by Deasy et al. ^22^.

Finally, our study focused on MIMIC-IV, a single-center U.S. database, which may limit generalizability. Monitoring practices, patient case mix, and measurement devices vary across institutions and countries. External validation on different datasets will be important to confirm our findings.

Beyond imputation alone, future research should also consider outlier handling. Reconstruction-based approaches that combine anomaly detection and imputation ^23^ could offer added robustness, especially in physiologic extremes. Similarly, methods developed for synthetic time-series generation ^24–26^ may be adaptable to imputation tasks, provided they maintain clinical plausibility.

## Conclusion

In summary, imputation effectiveness in ICU data depends as much on the structure of missingness as on the algorithm. SAITS and US-GAN were co-best overall, especially for random or short gaps, while accuracy declined at physiological extremes, where errors matter most. Despite its simplicity, linear interpolation remained competitive across many settings and robust to isolated sensor failures, with the advantages of transparency and low computational cost.

Benchmarks under MCAR systematically underestimate real-world error versus structured gaps (e.g., 1–3-hour blocks); MCAR should be viewed as an optimistic lower bound. Even so, differences within clinically relevant ranges were often modest, so simple, interpretable strategies may suffice when deployment, reproducibility, or explainability are priorities.

Finally, better imputation does not automatically yield better downstream models. This link warrants targeted study, ideally with tailored imputation by feature type (vitals, labs, therapies) to fairly assess how imputation quality propagates to algorithm performance.

## Supplementary Information

Supplementary Information.

## Data Availability

The data used in this study are from the MIMIC-IV database, which is publicly available to credentialed researchers via PhysioNet (https://physionet.org/content/mimiciv/2.2/). Access requires completion of the appropriate training and acceptance of the Data Use Agreement.

## Code availability

The code used to preprocess data, apply masking strategies, and train imputation models is available at the following GitHub repository: https://github.com/mpoette/icu-time-series-imputation. The implementation is based on the PyPOTS library ^27^ https://github.com/WenjieDu/PyPOTS and supports full reproducibility of the experiments.
