# Supplementary material for: Benchmarking imputation strategies for missing time-series data in critical care using real-world-inspired scenarios
Source: Sci Rep. 2026 Feb 10;16:8116. doi: 10.1038/s41598-026-39035-z (PMC12960818; doi:10.1038/s41598-026-39035-z)
Supplement: Supplementary file 1 — Supplementary Information. [file 41598_2026_39035_MOESM1_ESM.pdf]

Supplementary Material

Benchmarking Imputation Strategies for Missing Time-Series Data in Critical Care

S1. Distribution of Missing Data in ICU Time Series

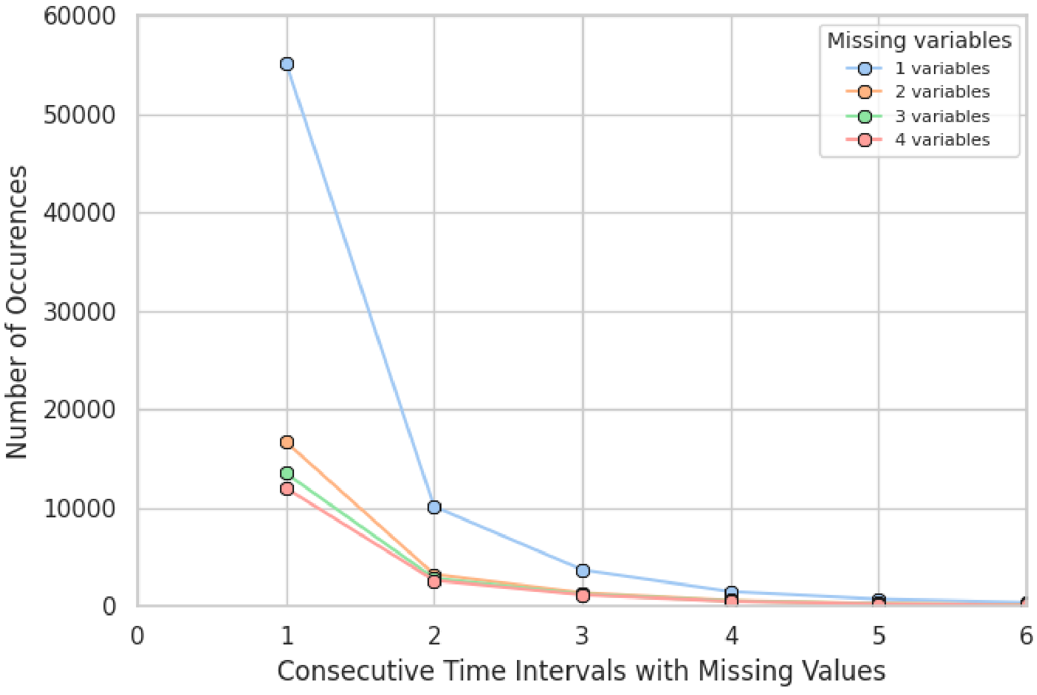

Figure S1: **Runs of consecutive missingness.** Number of occurrences as a function of the length of consecutive time intervals with missing values, stratified by how many variables are missing simultaneously (1–4). Short gaps are frequent; long runs are rare.

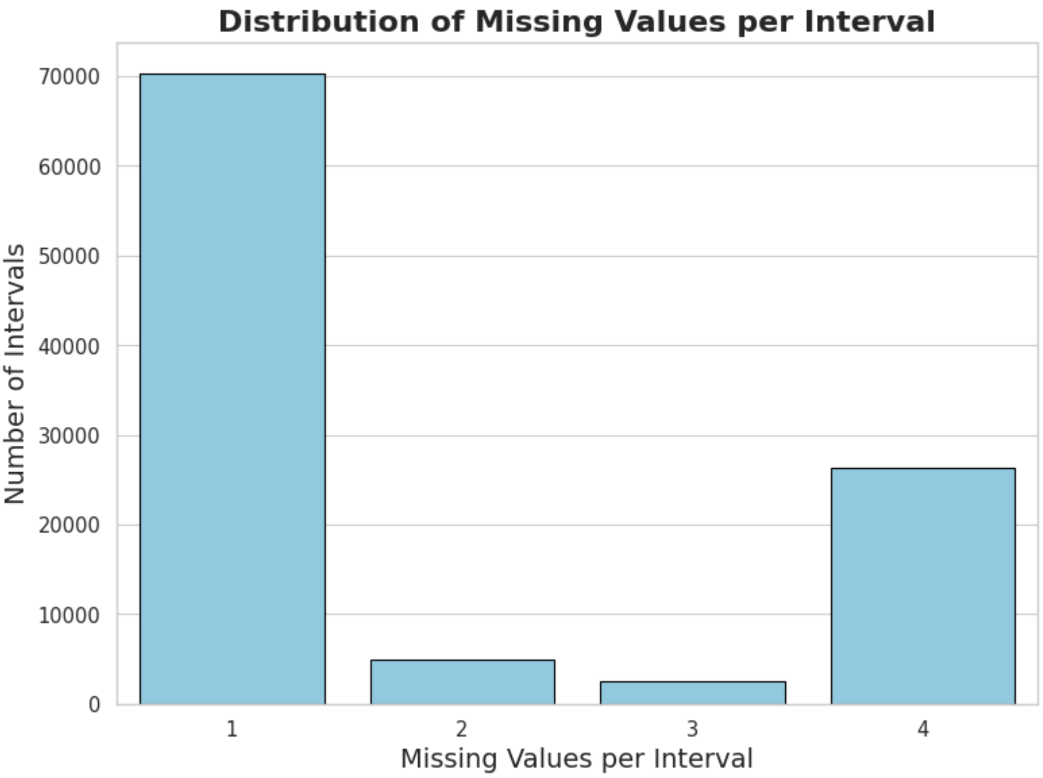

Figure S2: **Per-interval missingness.** Histogram showing the distribution of the count of missing variables per time interval. Most intervals have a single missing variable; intervals with 3–4 missing variables are less common.

S2. Training Protocol and Model Hyperparameters

Unless stated otherwise, training and evaluation follow the same data preprocessing and masking strategy as in the main text. Compared to the companion notebook, two changes apply for the experiments summarized in the paper:

- Hyperparameters were tuned with a grid search using a **20% validation split**.
- Deep models were trained for **200 epochs** (the notebook default is 50).

Common settings (from the notebook).

- Time horizon and dimensionality: $n_{\text{steps}} = 48,\ n_{\text{features}} = 7$.
- Device: `cuda` when available.
- Unless explicitly listed below, other options use the library defaults (PyPOTS or scikit-learn).

S2.1 Deep Imputation Models (PyPOTS)

Table S1: Key hyperparameters for deep models. Epochs set to 200 for the final experiments.

Model	Parameter	Value
SAITS	Layers	3
	d_{model}	256
	d_{ffn}	128
	Heads	4
	d_k, d_v	64, 64
	Dropout	0.10
	Other	Diagonal attention mask = `True`
BRITS	RNN hidden size	128
USGAN	RNN hidden size	128
GP-VAE	Latent size	64

Training. For SAITS, BRITS, USGAN, and GP-VAE, we trained for 200 epochs with early stopping as per library defaults (if enabled by the implementation). Optimizer, batch size, and learning rate follow PyPOTS defaults unless otherwise specified in the code. Model checkpoints were saved to the designated paths.

S2.2 Classical/Tree-Based Imputers

Table S2: Classical imputers configured as in the notebook; grid search used the same 20% validation set to select among candidate settings when applicable.

Imputer	Parameter	Value
MICE (scikit-learn `IterativeImputer`)	`max_iter`	30
	`random_state`	42
MissForest (`missforest` package)	`clf`	`LGBMClassifier` (LightGBM)
	`rgr`	`LGBMRegressor` (LightGBM)
	`max_iter`	10
	`initial_guess`	mean (continuous), mode (categorical)

Grid search details. We performed a discrete grid search on model-specific hyperparameters (e.g., SAITS depth/width, BRITS/USGAN hidden size candidates, GP-VAE latent size candidates, and classical imputer knobs where relevant), selecting the configuration with the best validation performance on the 20% hold-out set. Unless otherwise noted, model selection used mean absolute error (MAE) on the validation split; test metrics were computed only once on the final selected configuration.

S3. Reproducibility Notes

- Data were split at the *patient* level to avoid leakage across sets.
- Normalization and any mask generation were fit on the training set and applied to validation/test.
